# A Dual Promoter System to Monitor IFN-γ Signaling *in vivo* at Single-cell Resolution

**DOI:** 10.1247/csf.21052

**Published:** 2021-11-06

**Authors:** Taisei Tanaka, Yoshinobu Konishi, Hiroshi Ichise, Shinya Tsukiji, Michiyuki Matsuda, Kenta Terai

**Affiliations:** 1 Laboratory of Bioimaging and Cell Signaling, Research Center for Dynamic Living Systems, Graduate School of Biostudies, Kyoto University, Kyoto, Japan; 2 Medical Oncology, Dana-Farber Cancer Institute, Harvard Medical School, Boston, MA, USA; 3 Lymphocyte Biology Section, Laboratory of Immune System Biology, National Institute of Allergy and Infectious Diseases, National Institutes of Health, Bethesda, MD, USA; 4 Department of Nanopharmaceutical Sciences, Nagoya Institute of Technology, Nagoya, Japan; 5 Department of Pathology and Biology of Diseases, Graduate School of Medicine, Kyoto University, Kyoto, Japan; 6 Institute for Integrated Cell-Material Sciences, Kyoto University, Kyoto, Japan

**Keywords:** *in vivo* imaging, tumor microenvironment, interferon-gamma, dual promoter system

## Abstract

IFN-γ secreted from immune cells exerts pleiotropic effects on tumor cells, including induction of immune checkpoint and antigen presentation, growth inhibition, and apoptosis induction. We combined a dual promoter system with an IFN-γ signaling responsive promoter to generate a reporter named the interferon sensing probe (ISP), which quantitates the response to IFN-γ by means of fluorescence and bioluminescence. The integration site effect of the transgene is compensated for by the PGK promoter-driven expression of a fluorescent protein. Among five potential IFN-γ-responsive elements, we found that the interferon γ-activated sequence (GAS) exhibited the best performance. When ISP-GAS was introduced into four cell lines and subjected to IFN-γ stimulation, dose-dependency was observed with an EC_50_ ranging from 0.2 to 0.9 ng/mL, indicating that ISP-GAS can be generally used as a sensitive biosensor of IFN-γ response. In a syngeneic transplantation model, the ISP-GAS-expressing cancer cells exhibited bioluminescence and fluorescence signals in an IFN-γ receptor-dependent manner. Thus, ISP-GAS could be used to quantitatively monitor the IFN-γ response both *in vitro* and *in vivo*.

## Introduction

IFN-γ is a cytokine secreted by immune cells and has pleiotropic effects on anti-tumor immune response (Dighe *et al.*, 1994; Kaplan *et al.*, 1998). IFN-γ suppresses tumor growth by inducing cell death or arresting the cell cycle (Bromberg *et al.*, 1996; Chin *et al.*, 1997; Chin *et al.*, 1996; Fulda and Debatin, 2002). Besides IFN-γ increases MHC class I-dependent antigen presentation, and, thereby, susceptibility to CD8+ T cell-mediated killing (Meunier *et al.*, 2005). Conversely, IFN-γ has been reported to be involved in tumor immune escape (Beatty and Paterson, 2000; Taniguchi *et al.*, 1987). IFN-γ induces ligands such as PD-L1 and PD-L2 on cancer cells and contributes to their escape from T cells infiltrating the tumor (Dong *et al.*, 2002; Freeman *et al.*, 2000; Garcia-Diaz *et al.*, 2017). IFN-γ also increases the instability of the tumor cell genome (Takeda *et al.*, 2017), contributing to the ability of cancer cells to evade the immune system.

It is crucial to analyze the spatiotemporal spread of IFN-γ in the tumor microenvironment to understand the pleiotropic function of IFN-γ in tumors. Visualization of the IFN-γ production *in vivo* is helpful to understand its temporal changes and tissue specificity (Reynolds *et al.*, 2019). For this purpose, JAK-STAT signaling pathway can be a versatile indicator to visualize the response of cancer cells to IFN-γ. Nuclear translocation of STAT1 can be used as a surrogate marker for the initial response to IFN-γ stimulation (Samsonov *et al.*, 2013; Sanderson *et al.*, 2012; Thibaut *et al.*, 2020). When monitoring the expression of downstream transcription factors induced by STAT1, the bioluminescence system is also helpful for sensitive detection, and fluorescence can be used to monitor transcriptional activity at the cellular level (Hoekstra *et al.*, 2020). However, pre-existing biosensors are subject to the integration site effect or the copy number of the transgene; therefore, it would be necessary to standardize the fluorescence/bioluminescence signal to quantitatively compare the IFN-γ activity.

In this study, we combined a dual promoter system and an IFN-γ signal-responsive elements to create a probe that could compensate for the integration site effect and the copy number of the biosensor gene. Using this probe, we visualized the IFN-γ signaling in transplanted cancer *in vivo*.

## Material and Methods

### Plasmids

To generate the ISP (IFN-γ sensing probe) constructs, cDNA encoding mCherry-Akaluc (Iwano *et al.*, 2018) was inserted into the pPB piggyBac transposon vector (Yusa *et al.*, 2009), and the 3-phosphoglycerate kinase (PGK) promoter-driven Turquoise-GL-NLS gene cassette was inserted in the opposite direction with a stuffer sequence upstream of it (Adra *et al.*, 1987). Then, annealed oligoDNA containing a minimal promoter (MinP) and either four tandem repeats of the interferon γ-activated sequence (GAS) element (5'-AGTTTCATATTACTCTAAATC-3') (Hoekstra *et al.*, 2020), five tandem repeats of the interferon-stimulated response element (ISRE) (5'-TAGTTTCACTTTCCC-3') (Weihua *et al.*, 1997), five tandem repeats of the IRF1 elements (5'-TTTCCCCGAAA-3') (Pellegrini and Schindler, 1993), five tandem repeats of the Ly-6E elements (5'-ATTCCTGTAAG-3') (Pellegrini and Schindler, 1993) or five tandem repeats of the MIG elements (5'-CTTACTATAAA-3') (Pellegrini and Schindler, 1993) was cloned in front of the coding sequences of mCherry-Akaluc (Fig. S1). pCMV-mPBase was provided by Kosuke Yusa (Kyoto University, Kyoto, Japan).

### Tumor cell lines and culture conditions

The B16F10 melanoma cell line was purchased from the Cell Resource Center for Biomedical Research, Institute of Development, Aging and Cancer, Tohoku University (Sendai, Japan). The Panc02 mouse pancreatic ductal adenocarcinoma cell line was obtained from the National Institutes of Health (Bethesda, MD). The MC-38 mouse colon adenocarcinoma cell line was provided by Takeshi Setoyama and Tsutomu Chiba (Kyoto University). The Braf^V600E^ melanoma cell line (Braf^V600E^ melanoma) was provided by Caetano Reis e Sousa (The Francis Crick Institute, London, UK). Panc02 and MC-38 cells were maintained in DMEM high glucose (FUJIFILM Wako Pure Chemical Corporation, Osaka, Japan). B16F10 and Braf^V600E^ melanoma cells were cultured in RPMI 1640 (Thermo Fisher Scientific, Waltham, MA). All culture media were supplemented with 10% heat-inactivated FBS (SIGMA, St. Louis, MO), 100 U/mL penicillin, and 100 μg/mL streptomycin (penicillin-streptomycin mixed solution; Nacalai Tesque, Kyoto, Japan). Cell cultures were maintained at 37°C with 5% CO_2_.

### Stable cell lines

PiggyBac transposon plasmids encoding the ISP and pCMV-mPBase were cotransfected into Panc02 and MC-38 cells by using Lipofectamine 3000 (Thermo Fisher Scientific), and into B16F10 and Braf^V600E^ melanoma cells by using an Amaxa nucleofector system (Lonza, Basel, Switzerland). In Amaxa nucleofector electroporation, 5×10^5^ cells were pelleted and resuspended in 100 μl of homemade buffer (4 mM KCl, 10 mM MgCl_2_, 107 mM NaH_2_PO_4_, 13 mM Na_2_HPO_4_, 11 mM HEPES pH 7.75) with 5 μg of DNA, and then electroporated with program P-020 for B16F10 and T-023 for Braf^V600E^ melanoma. Two days after transfection, the cells were selected with 10 μg/mL blasticidin S (Invitrogen, San Diego, CA) for at least 1 week. For the cell lines for injection into mice, the fluorescence intensity of Turquoise-GL was normalized by cell sorting.

### Knockout cell lines

The *Gnaq*^–/–^ Braf^V600E^ melanoma was developed according to a previously reported method with modification (Konishi *et al.*, 2021). The CRISPR/Cas9 system was used to disrupt the expression of the *Ifngr1* genes. The targeted gRNA oligos (5'-CTGATGCTGTCTGCGAAGGT-3') were introduced into the pX459 vector (Addgene plasmid #48139; Cambridge, MA). Then, 5 μg of pX459 plasmid was transfected into 5×10^5^ cells using an Amaxa nucleofector system. Cells were subjected to single cell dilution cloning and examined for knockout by nucleotide sequencing (Fig. S2).

### In vitro characterization of reporter cells

Cells expressing ISPs were trypsinized and suspended in PBS containing 3% FBS, then filtrated through a 40 μm nylon cell strainer (Corning, Corning, NY) and analyzed with a FACS Aria IIIu cell sorter (Becton Dickinson, Franklin Lakes, NJ). The following combinations of lasers and emission filters were used for the detection of fluorescence: Turquoise-GL, a 407 nm laser and an ET470/24m filter (Chroma Technology Corporation, Bellows Falls, VT); mCherry, a 561 nm laser and a DF610/20 filter (Omega Optical, Brattleboro, VT). Cells were gated for size and granularity to exclude cell debris and aggregates. Data were analyzed using FlowJo software (Tree Star, Ashland, OR). The fluorescence intensity ratio of mCherry/Turquoise-GL was used to represent the promoter activity. To quantify the response to IFN-γ, the mean mCherry/Turquoise-GL value in the absence of IFN-γ was used for the normalization. To compensate for the actual expression level of the fluorescent protein and the acquired fluorescence intensity, the sensitivity of the detector was adjusted so that the average fluorescence intensity of mCherry and Turquoise-GL was about 100 times higher than that in cells not expressing the fluorescent protein.

### Cell proliferation assay

Cell proliferation was assessed by counting the number of Turquoise-GL-labeled nuclei by time-lapse imaging with widefield microscopy. Cells cultured on glass-base 96-well plates were observed under an IX83 inverted microscope (Olympus, Tokyo, Japan) equipped with a UPlanSApo 40x/0.95 objective lens (Olympus), a PRIME scientific CMOS camera (Photometrics, Tucson, AZ), a Spectra-X light engine (Lumencor, Beaverton, OR), an IX2-ZDC laser-based autofocusing system (Olympus), and an incubation chamber (Tokai Hit, Fujinomiya, Japan). The filters and dichroic mirrors used for time-lapse imaging were as follows: 430/24 (Olympus) excitation filters, XF2034 (455DRLP) (Omega Optical) dichroic mirrors, and FF01-483/32 (Semrock, Rochester, NY) emission filters. Image acquisition and analysis were carried out with MetaMorph (Molecular Devices Japan, Tokyo, Japan) and ImageJ (National Institute of Health), respectively.

### Mice

B6 albino mice were obtained from Charles River Laboratories Japan (Yokohama, Japan). Mice were housed in a specific pathogen-free facility and received a routine chow diet and water *ad libitum*. The animal protocols were reviewed and approved by the Animal Care and Use Committee of Kyoto University Graduate School of Medicine (MedKyo20081).

### Tumor cell injections

Cells were harvested by trypsinization, washed three times with PBS, and injected subcutaneously into the flank of recipient mice at 2×10^5^ cells in 100 μl of 50% Matrigel (Corning) in PBS. Tumor size was quantified by multiplying the longest diameter, its perpendicular, and its thickness.

### Processing of tumor tissue and flow cytometry

Tumors were dissected into pieces and digested with Collagenase Type IV (200 U/mL) (Worthington Biochemical Corporation, Lakewood, NJ) and DNase I (10 U/mL) (Roche Diagnostics, Indianapolis, IN) for 30 minutes at 37°C. After washing of cells with PBS, the fluorescence intensity of individual cells was determined with a FACS Aria IIIu.

### Bioluminescence imaging

Mice bearing tumors were anesthetized with 2% isoflurane (FUJIFILM Wako Pure Chemical Corporation) inhalation (the O_2_ to air gas ratio was over 95%) and placed on a custom-made heating plate in the supine position. Bioluminescent images were acquired using a MIIS system (Molecular Devices Japan) equipped with an iXon Ultra EMCCD camera (Oxford Instruments, Belfast, UK) and a lens (MDJ-G25F095, φ16 mm, F: 0.95; Tokyo Parts Center, Saitama, Japan). Akalumine-HCl, also called TokeOni, was obtained from Kurogane Kasei (Nagoya, Japan) and used as the substrate of Akaluc. Five minutes after the start of image acquisition, 100 μL of 5 mM AkaLumine-HCl was administered (i.p.). Images were acquired under the following conditions: binning, 4; EM gain, 0. Acquisition of bioluminescent images was repeated every 30 seconds, and the maximum bioluminescent intensity during the imaging was adopted in each mouse. Image acquisition and analysis were carried out with MetaMorph and ImageJ, respectively.

### Intravital imaging by two-photon excitation microscopy

Intravital imaging was performed as previously described with some modifications (Konishi *et al.*, 2021; Yamauchi *et al.*, 2016). In brief, mice were anesthetized with 2% isoflurane inhalation (the O_2_ to air gas ratio was 80:20) and placed in the prone position on an electric heating pad. The body temperature was maintained at 36.5°C. The skin flap was then placed on a coverglass.

Mice were observed with an FV1000MPE-IX-83 inverted microscope (Olympus) equipped with a UPLSAPO 30XS/1.05 numerical aperture (NA) silicon-immersion objective lens (Olympus) and an InSight DeepSee Ultrafast laser (Spectra Physics, Mountain View, CA). The excitation wavelength for Turquoise-GL was 840 nm, and that for mCherry was 1040 nm. Fluorescent images were acquired with two different detector channels using the following filters and mirrors: an infrared (IR)-cut filter, BA685RIF-3 (Olympus), two dichroic mirrors, DM505 and DM570 (Olympus), and three emission filters, FF01-425/30 (Semrock) for the second harmonic generation channel (SHG), BA460-500 (Olympus) for Turquoise-GL, and BA575-630 (Olympus) for mCherry. The microscope was equipped with a two-channel GaAsP detector unit and two multialkali detectors. FLUOVIEW software version 4.1a (Olympus) was used to control the microscope and to acquire images, which were saved in the multilayer 16-bit tagged image file format.

For the measurement of ISP-GAS activity *in vivo*, regions of interest (ROIs) were set at nuclei in a single plane by Turquoise-GL signal with Cellpose (Stringer *et al.*, 2021). The integrated mCherry signal in the ROI was divided by the integrated Turquoise-GL intensity in the ROI and shown as ISP-GAS activity.

### Statistical analysis

The cell EC_50_ was calculated by undertaking a two-parameter logistic curve fitting of the normalized dose-response curve using Python (version 3.7). All statistical analyses were performed using Python. No statistical analysis was used to predetermine the sample size. Welch’s t-test was used to evaluate statistically significant differences. The p-values less than 0.05 were considered statistically significant.

For all the box plots, the edges on the box plots indicate the first and third quartiles, with the line in the middle being the mean. The whiskers on the box plots extend another 1.5 x the interquartile range from the edges of the boxes, respectively.

## Results and Discussion

### Generation of the IFN-γ sensing probe, ISP

**Fig. 1:**
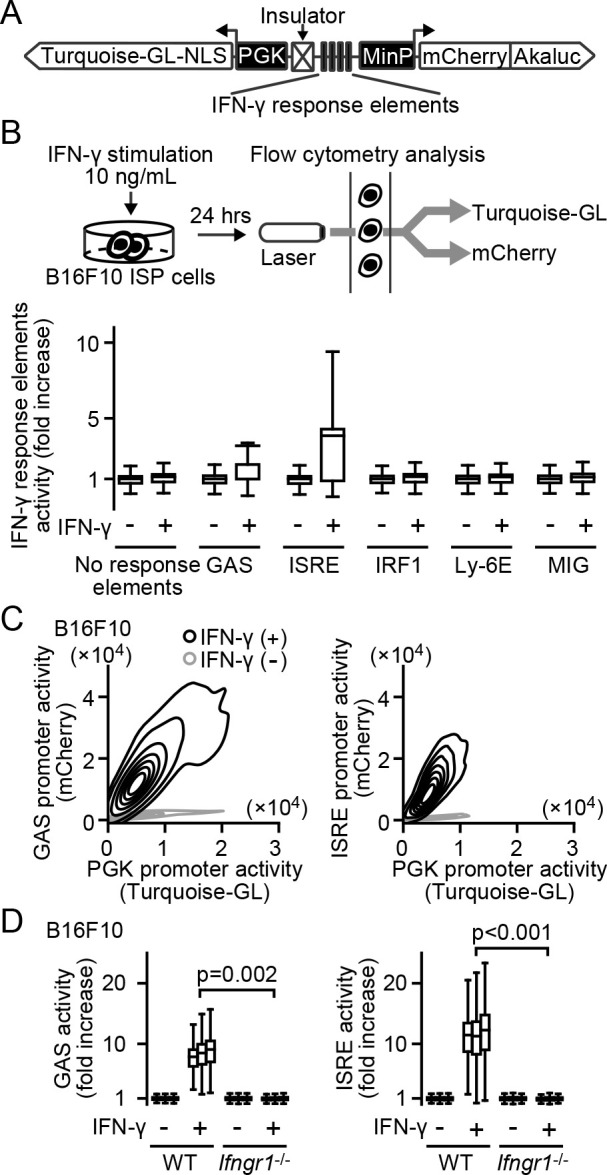
Generation of the IFN-γ sensing probes, ISP. (A) Schematic representation of the ISP. One of the five IFN response elements is employed in each construct. (B and C) B16F10 cells transiently (B) or stably (C) expressing the ISPs. Cells were cultured in the presence or absence of 10 ng/mL IFN-γ for 24 hours, and analyzed by flow cytometry. The mCherry/Turquoise-GL fluorescence intensity ratios were normalized to the mean in the absence of IFN-γ. The normalized values are used as the promoter activity and shown in the box plot (n=1) (B). Representative flow cytometry results are shown as 12.5% probability contour plots (C). (D) ISP activity in B16F10 WT or *Ifngr1*^–/–^ cells. Cells were cultured in the presence or absence of 10 ng/mL IFN-γ for 24 hours, and measured the GAS or ISRE activities as shown in panel B. The p-values of Welch’s t-test were calculated by sample means (n=3).

To monitor the response to IFN-γ in tumor cells, the probe must satisfy two requirements. First, the cDNA of the probe must be stably expressed in tumor cells. Second, the integration site effect on the IFN-γ-dependent transcriptional activity must be compensated. For this, we generated a dual promoter plasmid named ISP. This reporter transcribes an mCherry-Akaluc fusion protein under an IFN-γ signal-responsive promoter (Fig. 1A). In addition, a Turquoise-GL protein with a nuclear localization signal (NLS) is expressed under the ubiquitous promoter, PGK, to compensate for the locus effect. With this, the promoter activity can be measured by the mCherry/Turquoise-GL fluorescence intensity ratio. We screened five promoter elements that may respond to IFN-γ in B16F10 melanoma cells. Among them, GAS (interferon γ-activated sequence) and ISRE (interferon-stimulated response element) responded to the INF-γ stimulation (Fig. 1B). As anticipated, cells expressing ISP-GAS or ISP-ISRE showed linear correlation between the mCherry and Turquoise-GL intensities (Fig. 1C), suggesting effective compensation of the integration site effect in the population. We next validated the specificity of the ISP-GAS and ISP-ISRE by using IFNGR1, the IFN-γ receptor, knockout cell line (Fig. 1D). Collectively, the ISPs can monitor the IFN-γ response with little or no integration site effect.

### Dose responses of ISP-GAS and -ISRE

**Fig. 2:**
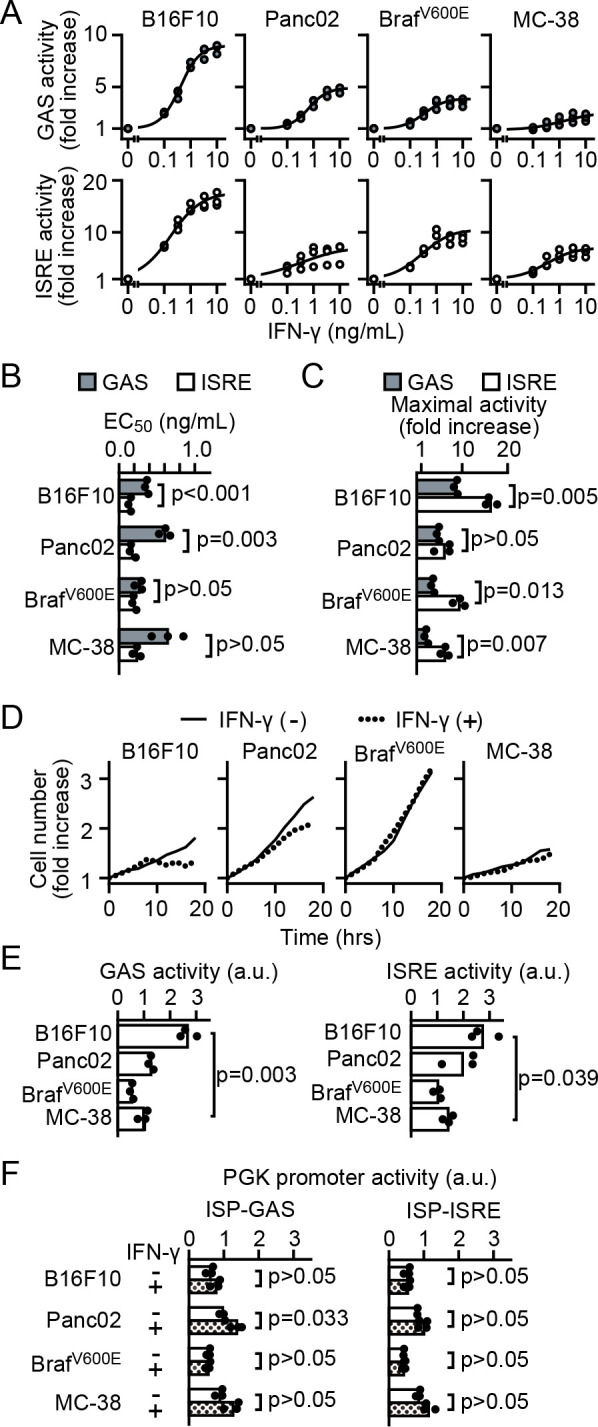
Dose responses of ISP-GAS and -ISRE. (A to C) Dose-response curves of ISP-GAS and ISP-ISRE. (A) Cells expressing ISP-GAS or ISP-ISRE were plated on 48-well dishes and cultured in the absence or presence of 0.1 to 10 ng/mL IFN-γ for 24 hours. GAS or ISRE activity was analyzed by flow cytometry. GAS or ISRE activity is normalized with the mean value without IFN-γ stimulation and plotted against the IFN-γ concentration. Three independent experiments are fitted to the Hill equation and shown by the solid lines. EC_50_ (B) and maximal activity (C) are deduced from the data in (A). Results are shown from three independent experiments. (D) Proliferation of B16F10, Panc02, Braf^V600E^, and MC-38 cells in the presence or absence of 10 ng/mL IFN-γ for the indicated hours. (E) Absolute ratios of mCherry/Turquoise-GL, ISP activity, with IFN-γ at 10 ng/mL for 24 hours, referred from panel A. (F) The intensities of Turquoise-GL representing PGK promoter. The p-values of Welch’s t-test were calculated by sample means (n=3).

To validate the versatility of ISP, we expressed ISP in four C57BL/6-derived cell lines, B16F10 melanoma cells, Panc02 pancreatic adenocarcinoma cells, Braf^V600E^ melanoma cells, and MC-38 colon adenocarcinoma cells, and examined the dose response to IFN-γ (Fig. 2A). In all cell lines, clear dose-response curves were obtained by both ISP-GAS and ISP-ISRE. The EC_50_ values were between 0.2 and 0.9 ng/mL in ISP-GAS-expressing cell lines and between 0.1 and 0.3 ng/mL in ISP-ISRE-expressing cell lines (Fig. 2B). The fold increase of the mCherry/Turquoise-GL fluorescence intensity ratio was always smaller in the cell line harboring ISP-GAS than in that harboring ISP-ISRE (Fig. 2C), demonstrating that the sensitivity of ISP-ISRE, specifically *in vitro* situation, is higher than that of ISP-GAS.

IFN-γ directly inhibits tumor cell growth and promotes tumor cell apoptosis (Trubiani *et al.*, 1994) via the JAK-STAT pathway (Chin *et al.*, 1997; Detjen *et al.*, 2001). We asked whether the response detected by ISP correlates with the biological susceptibility to IFN-γ. We found that B16F10 and Panc02, but not Braf^V600E^ or MC-38, showed growth retardation after 10 hours of IFN-γ treatment (Fig. 2D), as reported previously (Gerber *et al.*, 2013; Mazzolini *et al.*, 2003; Zoller, 1988). What was responsible for this difference? We found that the mCherry/Turquoise-GL fluorescence intensity ratios of ISP-GAS and ISP-ISRE were higher in IFN-γ-stimulated B16F10 and Panc02 than in Braf^V600E^ or MC-38 cells (Fig. 2E), when the Turquoise-GL signals were similar among cell lines (Fig. 2F). These results demonstrate that the absolute transcriptional activity of B16F10 and Panc02 with IFN-γ stimulation is higher than those of Braf^V600E^ or MC-38 cells. The difference of the susceptibility to IFN-γ, which is regulated by intracellular signaling pathway, may explain the difference in this growth retardation. Although the difference was marginal, the response detected by ISP-GAS may have been suitable for the prediction of the biological response.

### ISP-GAS, an IFN-γ-specific reporter in vivo

**Fig. 3:**
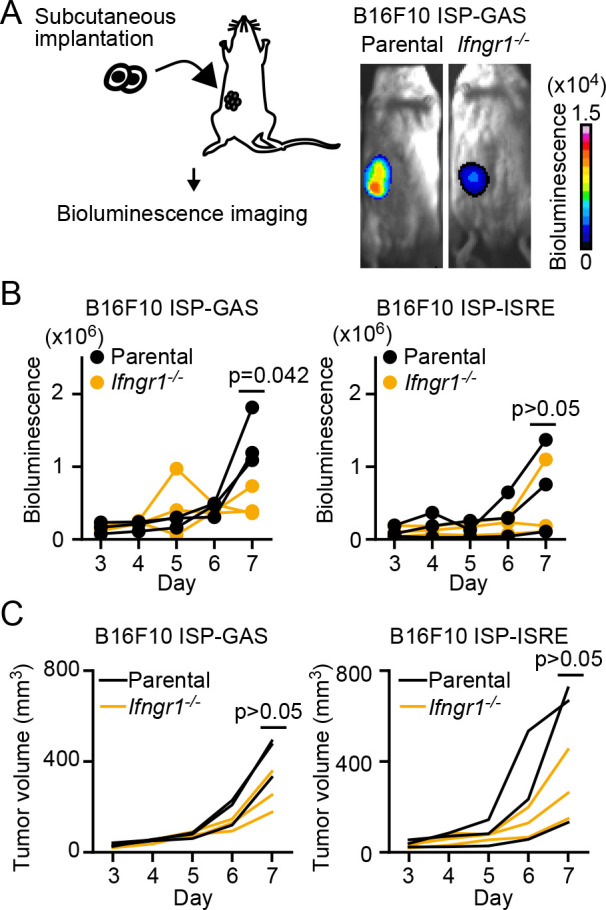
ISP-GAS, an IFN-γ specific reporter *in vivo*. (A to C) ISP-GAS and ISP-ISRE *in vivo*. B6 albino mice were subcutaneously implanted with parental or *Ifngr1*^–/–^ B16F10 cells expressing ISP-GAS. Shown here are the representative merged images of the bright field and the bioluminescence image at 7 days after implantation (A). The bioluminescence intensity (B) and the tumor volume (C) were analyzed from Day 3 to Day 7 after implantation. p-values were calculated at 7 days after implantation by Welch’s t-test.

We next examined whether ISP-GAS could be used to monitor the IFN-γ response *in vivo*. In ISPs, mCherry is fused to the ultrasensitive bioluminescent protein Akaluc (Fig. 1A). Therefore, GAS-dependent transcription was monitored by Akaluc bioluminescence in B6 albino mice implanted with ISP-GAS-expressing B16F10 cells (Fig. 3A). As a control, *Ifngr1*^–/–^ B16F10 cells were also challenged. Seven days after inoculation, the bioluminescence signal was higher in the parental B16F10 cells than in the *Ifngr1*^–/–^ cells (Fig. 3B, left panel). Meanwhile, the difference was not reproducible when ISP-ISRE was used (Fig. 3B, right panel). The difference in the tumor size was not significant between the parent and *Ifngr1*^–/–^ cells (Fig. 3C). Thus, we concluded that ISP-GAS specifically monitors the IFN-γ signaling pathway *in vivo*.

The reason that we failed to detect a significant difference in the ISP-ISRE response between the parent and *Ifngr1*^–/–^ cells may be ascribable to the previous observation that ISRE is also activated by type-I IFNs including IFN-α, IFN-β, and IFN-ω (Khiar *et al.*, 2017; Tanaka *et al.*, 1993; Uccellini and Garcia-Sastre, 2018). If so, ISP-ISRE could be used as a pan-IFN reporter including the type-II IFN, IFN-γ, after further characterization.

### Enhanced IFN-γ signaling in Gnaq^–/–^ tumor cells in vivo

**Fig. 4:**
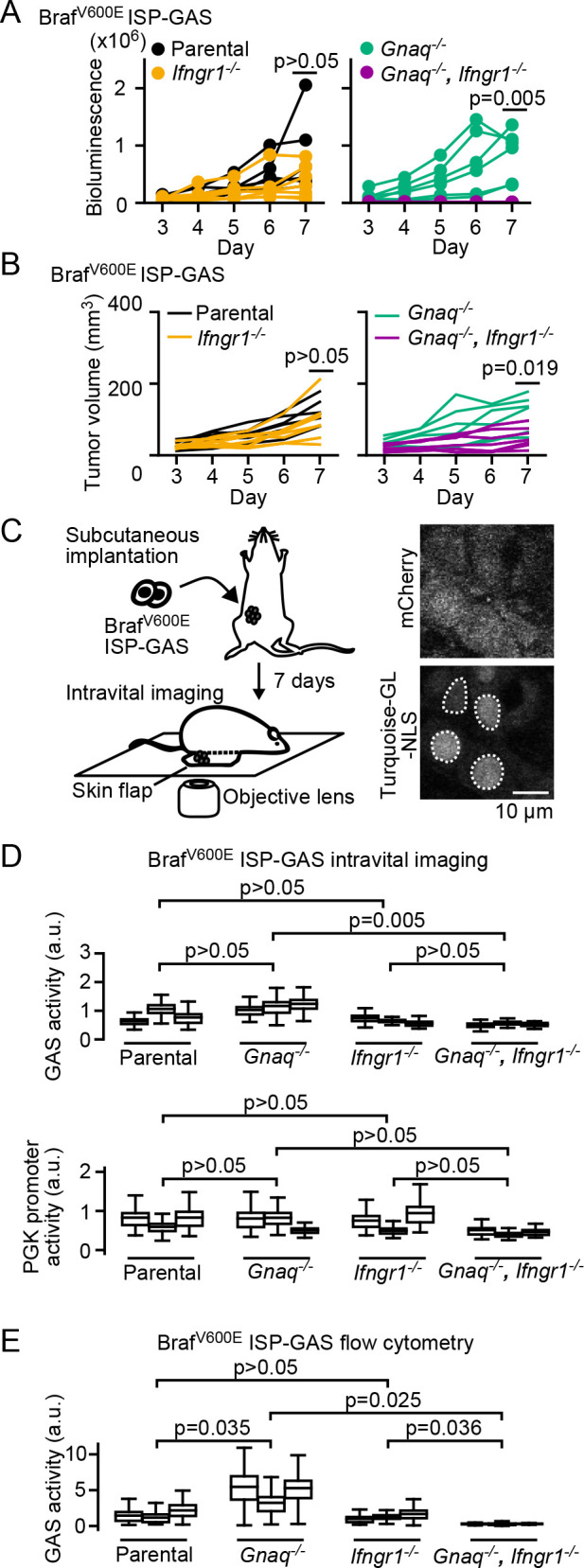
Enhanced IFN-γ signaling in *Gnaq*^–/–^ tumor cells *in vivo*. (A and B) ISP-GAS in Braf^V600E^ melanoma cells. B6 albino mice were subcutaneously implanted with parental, *Ifngr1*^–/–^, *Gnaq*^–/–^, or *Ifngr1*^–/–^/*Gnaq*^–/–^ Braf^V600E^ melanoma cells expressing ISP-GAS. The bioluminescence intensity (A) and the tumor volume (B) were measured from Day 3 to Day 7. The data represent six or seven mice from three independent experiments. p-values were calculated with values at 7 days after implantation by Welch’s t-test. (C and D) ISP-GAS Braf^V600E^ melanoma cells under two-photon microscopy at 7 days after tumor implantation. For imaging of ISP-GAS Braf^V600E^ melanoma cells implanted subcutaneously, the tumor tissues were exposed by the skin-flap method and observed under an inverted two-photon excitation microscope (left panel). Shown here are the representative images of the mCherry and Turquoise-GL-NLS of ISP-GAS parental cells (right panel). ROIs (white dotted line) were set at the nuclei (C). GAS activity was calculated by dividing the mCherry intensity by the Turquoise-GL intensity in the ROIs and shown in the box plots (D). The Turquoise-GL intensity in the ROIs also shown in the box plots. (E) Flow cytometry analysis of the tumor in (D). The GAS activity was calculated by dividing the mCherry intensity by the Turquoise-GL intensity and shown in the box plots. All the p-values of Welch’s t-test were calculated by sample means (n=3).

We recently reported that Braf^V600E^ melanoma cells maintain an immunocompromised microenvironment by means of thromboxane A2-mediated activation of the GqPCR, Gq protein-coupled receptors, pathway (Konishi *et al.*, 2021). Activated GqPCR triggers the secretion of prostaglandin E2 (PGE2) from tumor cells, which, in turn, suppress the recruitment of NK cells, a principal producer of IFN-γ in the tumor microenvironment (Zelenay *et al.*, 2015). We therefore examined the effect of the suppressed GqPCR signaling pathway on the IFN-γ response by the knockout of Gnaq, the Gq protein alpha subunit. In the parental Braf^V600E^ melanoma cells, the bioluminescence signal of ISP-GAS was higher than in the *Ifngr1*^–/–^ cells, although the difference was not statistically significant (Fig. 4A, left). On the other hand, *Gnaq*^–/–^ cells exhibited a markedly higher bioluminescence signal of ISP-GAS than *Gnaq*^–/–^ *Ifngr1*^–/–^ double-knockout cells (Fig. 4A, right), supporting the notion that IFN-γ is abundant in the tumor microenvironment. However, the difference in tumor volume would affect the bioluminescence signal (Fig. 4B), which motivated us to analyze the IFN-γ signaling at single-cell resolution.

Against our expectation, we did not observe a significant difference in bioluminescence between the parental and *Gnaq*^–/–^ cells. Thus, we next analyzed tumor cells at single-cell resolution using two-photon microscopy (Fig. 4C, D) and flow cytometry (Fig. 4E). As anticipated, the GAS activity, mCherry/Turquoise-GL ratio, in *Gnaq*^–/–^ cells was significantly higher than that in parental cells, supporting the abundance of IFN-γ in *Gnaq*^–/–^ cells.

Why did the *Ifngr1*^–/–^ cells show higher GAS activities than *Gnaq*^–/–^ *Ifngr1*^–/–^ double-knockout cells in flow cytometry analysis? We suppose that JAK-STAT pathway could be also activated via other cytokines rather than IFN-γ (O’Shea and Plenge, 2012; Salas *et al.*, 2020), though it is not dominant for ISP-GAS. Since we have demonstrated that the removal of Gq protein transforms the microenvironment into tumor-killing immune microenvironment (Konishi *et al.*, 2021), the microenvironment would be abundant in cytokines such as IL-12b and IL-10 (Zelenay *et al.*, 2015).

What is the advantage of ISP-GAS in comparison to the preceding probes of the IFN-γ signaling pathway (Koster and Hauser, 1999; Reynolds *et al.*, 2019; Samsonov *et al.*, 2013; Sanderson *et al.*, 2012; Stifter *et al.*, 2019; Taniguchi *et al.*, 1987; Thibaut *et al.*, 2020; Uccellini and Garcia-Sastre, 2018)? First, because of the constitutive Turquoise-GL expression, the dual promoter system of ISP-GAS allows us to validate the IFN-γ signaling at single-cell resolution. The intensity of bioluminescence alone could be affected by the number of cells in the tumor mass, the depth of the tumor below the body surface, and the technical reproducibility of the substrate injection (Baba *et al.*, 2007). These drawbacks would be minimized by using ISP-GAS with FACS analysis and intravital imaging, as shown in Fig. 4D and 4E. Second, we can compare the IFN-γ signaling in a variety of cell lines *in vivo*. In tumor microenvironments, several epigenetic modifiers have been reported, including IFNs (Ivashkiv, 2018; Ivashkiv and Donlin, 2014), which would alter the expression of luciferase and/or Turquoise-GL-NLS. Indeed, in our case, the intensity of Turquoise-GL-NLS was decreased in *Gnaq*^–/–^ *Ifngr1*^–/–^ double-knockout Braf^V600E^ cells expressing ISP-GAS at 7 days after tumor implantation, while the mCherry/Turquoise-GL ratio was still low (Fig. 4D). Our dual promoter system successfully compensated for the epigenetic status and integration site effect. Third, ISP-GAS facilitates monitoring of the IFN-γ signaling in living mice. Fluorescent protein-tagged Stat1 is also widely used for detecting the IFN-γ signaling, because of its fast kinetics (Thibaut *et al.*, 2020). However, it would be time-consuming to identify the appropriate spatio-temporal window under microscopy. A hybrid combining the advantages of bioluminescence and fluorescence will help to identify them.

Collectively, our results demonstrated that ISP-GAS allows us to monitor the activity of the IFN-γ signaling pathway at single-cell resolution and thereby monitor immune activity in the tumor microenvironment.

## Abbreviations

IFN-γ: interferon-gamma
ISP: interferon sensing probe
