# Supplementary material for: A Dual Promoter System to Monitor IFN-γ Signaling in vivo at Single-cell Resolution
Source: Cell Struct Funct. 2021 Nov 6;46(2):103–11. doi: 10.1247/csf.21052 (PMC10511040; doi:10.1247/csf.21052)
Supplement: Supplementary file 1 — Fig. S1 [file csf_46_21052_1.pdf]

Figure S1

Sequences of ISP plasmid

IFN-γ Response elements

IFN-γ response element	Sequence
GAS	AGTTTCATATTACTCTAAATCAGTTTCATATTACTCTAAATCAGTTTCATATTACTCTAAATCAGTTTCATATTACTCTA AATC
ISRE	TAGTTTCACTTTCCCTAGTTTCACTTTCCCTAGTTTCACTTTCCCTAGTTTCACTTTCCCTAGTTTCACTTTCCC
IRF1	TTTCCCCGAAATTTCCCCGAAATTTCCCCGAAATTTCCCCGAAATTTCCCCGAAA
Ly-6E	ATTCCTGTAAGATTCCTGTAAGATTCCTGTAAGATTCCTGTAAGATTCCTGTAAG
MIG	CTTACTATAAACTTACTATAAACTTACTATAAACTTACTATAAACTTACTATAAA

CTAAATTGTAAGCGTTAATATTTTGTTAAAATTCGCGTTAAATTTTTGTTAAATCAGCTCATTTTTTAACCAATAGGCCGAAATCGGCAAAATC
CCTTATAAATCAAAAGAATAGACCGAGATAGGGTTGAGTGTTGTTCCAGTTTGGAACAAGAGTCCACTATTAAAGAACGTGGACTCCAACGTCA
AAGGGCGAAAAACCGTCTATCAGGGCGATGGCCCACTACGTGAACCATCACCCTAATCAAGTTTTTTGGGGTCGAGGTGCCGTAAAGCACTAAA
TCGGAACCCTAAAGGGAGCCCCCGATTTAGAGCTTGACGGGGAAAGCCGGCGAACGTGGCGAGAAAGGAAGGGAAGAAAGCGAAAGGAGCGGGC
GCTAGGGCGCTGGCAAGTGTAGCGGTCACGCTGCGCGTAACCACCACACCCGCCGCGCTTAATGCGCCGCTACAGGGCGCGTCCCATTCGCCAT
TCAGGCTGCGCAACTGTTGGGAAGGGCGATCGGTGCGGGCCTCTTCGCTATTACGCCAGCTGGCGAAAGGGGGATGTGCTGCAAGGCGATTAAG
TTGGGTAACGCCAGGGTTTTCCCAGTCACGACGTTGTAAAACGACGGCCAGTGAGCGCGCGTAATACGACTCACTATAGGGCGAATTGGGGCGC
GCCATTCTAGATTAACCCTAGAAAGATAGTCTGCGTAAAATTGACGCATGCATTCTTGAAATATTGCTCTCTCTTTCTAAATAGCGCGAATCCG
TCGCTGTGCATTTAGGACATCTCAGTCGCCGCTTGGAGCTCCCGTGAGGCGTGCTTGTCAATGCGGTAAGTGTCACTGATTTTGAACTATAACG
ACCGCGTGAGTCAAAATGACGCATGATTATCTTTTACGTGACTTTTAAGATTTAACTCATACGATAATTATATTGTTATTTCATGTTCTACTTA
CGTGATAACTTATTATATATATATATTTTCTTGTTATAGATATCAACTAGAATGCTAGCATGGGCCCATCTCGACATTGATTATTGACTAGTCCAT
AGAGCCCACCGCATCCCCAGCATGCCTGCTATTGTCTTCCCAATCCTCCCCCTTGCTGTCCTGCCCCACCCCACCCCCCAGAATAGAATGACAC
CTACTCAGACAATGCGATGCAATTTCCTCATTTTATTAGGAAAGGACAGTGGGAGTGGCACCTTCCAGGGTCAAGGAAGGCACGGGGGAGGGGC
AAACAACAGATGGCTGGCAACTAGAAGGCACAGTCGAGGCTGATCAGCGAGCTCTAGATCATCGATGCATCTCGACTTTTAATTTCGGGTATAT
TTGAGTGGAATGAGTTCTTCAATCGTAGTTTTGACTAACTTGCCATTCATTTCTATTAACACAAAACAATCTGGTGCATAGTCTGAAATCAACT
CCCTACACATACCACAAGGACTTACCACTCGAATACTTCTATCTACTTCGTCAGAATAAGGGTGTCTAACAGCTACAATCGTGTCAAAATCCTT
TTGTCCATTCGAAACTGCACTACCAATCGCAATGGCTTCTGCACAAACAGTTACTCGTCCTATATACGCTTCAATATGTACTGCCGAAATGATT
TCTCCTGTTTTCGTACGAATTGCCGCTCCCACATGATGTTTATTATCCTCATAAAGCATTGTAATCTTCTCTGTCGCTACTTCTACTAATTCTA
GATCTTGTTGAGAAATGTTAAATGTTTTCATGACCATGGTATTATCGTGTTTTTCAAAGGAAAACCACGTCCCCGTGGTTCGGGGGGCCTAGAC
GTTTTTTAACCTCGACTAAACACATGTAAAGCATGTGCACCGAGGCCCCAGATCAGATCCCATACAATGGGGTACCTTCTGGGCATCCTTCAGC
CCCTTGTTGAATACGCTTGAGGAGAGCCATTTGACTCTTTCCACAACTATCCAACTCACAACGTGGCACTGGGGTTGTGCCGCCTTTGCAGGTG
TATCTTATACACGTGGCTTTTGGCCGCAGAGGCACCTGTCGCCAGGTGGGGGGTTCCGCTGCCTGCAAAGGGTCGCTACAGACGTTGTTTGTCT
TCAAGAAGCTTCCAGAGGAACTGCTTCCTTCACGACATTCAACAGACCTTGCATTCCTTTGGCGAGAGGGGAAAGACCCCTAGGAATGCTCGTC
AAGAAGACAGGGCCAGGTTTCCGGGCCCTCACATTGCCAAAAGACGGCAATATGGTGGAAAATAACATATAGACAAACGCACACCGGCCTTATT
CCAAGCGGCTTCGGCCAGTAACGTTAGGGGGGGGGGGCGGAATTGATCCCGCTAGAGGGAAACCGTTGCTAGCTTAAGTTAGTTAACAATTGTT
ACCGCGGCCCGTCGACTTACACCTTACGCTTCTTCTTAGGTCTAGACAGCTCGTCCATGCCGAGAGTGATCCCGGCGGCGGTCAAGAACTCCAG
CAGGACCATGTGATCGCGCTTCTCGTTGGGGTCTTTGCTTAAGGCGGACTGGGTGCTCAGGTAGTGGTTGTCGGGCAGCAGCACGGGGCCGTCG
CCGATGGGGGTGTTCTGCTGGTAGTGGTCGGCGAGCTGCACGCCGCCGTCCTCGATGTTGTGGCGGATCTTGAAGTTGGCCTTGATGCCGTTCT
TCTGCTTGTCGGCGGTGATATAGACGTTCCCGCTGATGTAGTTGTACTCCAGCTTGTGCCCCAGGATGTTGCCGTCCTCCTTGAAGTCGATGCC
CTTCAGCTCGATGCGGTTCACCAGGGTGTCGCCCTCGAACTTCACCTCGGCGCGGGTCTTGTAGTTGCCGTCGTCCTTGAAGAAGATGGTGCGC
TCCTGGACGTAGCCTTCGGGCATGGCGGACTTGAAGAAGTCGTGCTGCTTCATGTGGTCGGGGTAGCGGGCGAAGCACTGCACGCCCCAGGACA
GGGTGGTCACGAGGGTGGGCCAGGGCACGGGCAGCTTGCCGGTGGTGCAGATGAACTTCAGGGTCAGCTTGCCGTAGGTGGCATCGCCCTCGCC
CTCGCCGGACACGCTGAACTTGTGGCCGTTTACGTCGCCGTCCAGCTCGACCAGGATGGGCACCACCCCGGTGAACAGCTCCTCGCCCTTGCTC
ACCATGGCGTCGCGAAGGTCGAAAGGCCCGGAGATGAGGAAGAGGAGAACAGCGCGGCAGACGTGCGCTTTTGAAGCGTGCAGAATGCCGGGCC
TCCGGAGGACCTTCGGGCGCCCGCCCCGCCCCTGAGCCCGCCCCTGAGCCCGCCCCCGGACCCACCCCTTCCCAGCCTCTGAGCCCAGAAAGCG
AAGGAGCAAAGCTGCTATTGGCCGCTGCCCCAAAGGCCTACCCGCTTCCATTGCTCAGCGGTGCTGTCCATCTGCACGAGACTAGTGAGACGTG
CTACTTCCATTTGTCACGTCCTGCACGACGCGAGCTGCGGGGCGGGGGGGAACTTCCTGACTAGGGGAGGAGTAGAAGGTGGCGCGAAGGGGCC
ACCAAAGAACGGAGCCGGTTGGCGCCTACCGGTGGATGTGGAATGTGTGCGAGGCCAGAGGCCACTTGTGTAGCGCCAAGTGCCCAGCGGGGCT
GCTAAAGCGCATGCTCCAGACTGCCTTGGGAAAAGCGCCTCCCCTACCCGGTAGAACATATGCTCGACCGCGCCGCCCCGGGCGGCTCGCTGCT
GCCCCCTAGCGGGGGAGGGACGTAATTACATCCCTGGGGGCTTTGGGGGGGCTCGATAGTGAATTCGGTCACC IFN-γ Response elements
CTCGAGGATATCAAGATCTGGCCTCGGCGGCCAAGCTTAGACACTAGAGGGTATATAATGGAAGCTCGACTTCCAGCTTGGCAATCCGGTACTG
TTGGTAAAGCCAGCGGCCGCCACCATGGTGAGCAAGGGCGAGGAGGATAACATGGCCATCATCAAGGAGTTCATGCGCTTCAAGGTGCACATGG
AGGGCTCCGTGAACGGCCACGAGTTCGAGATCGAGGGCGAGGGCGAGGGCCGCCCCTACGAGGGCACCCAGACCGCCAAGCTGAAGGTGACCAA
GGGTGGCCCCCTGCCCTTCGCCTGGGACATCCTGTCCCCTCAGTTCATGTACGGCTCCAAGGCCTACGTGAAGCACCCCGCCGACATCCCCGAC
TACTTGAAGCTGTCCTTCCCCGAGGGCTTCAAGTGGGAGCGCGTGATGAACTTCGAGGACGGCGGCGTGGTGACCGTGACCCAGGACTCCTCCC
TGCAGGACGGCGAGTTCATCTACAAGGTGAAGCTGCGCGGCACCAACTTCCCCTCCGACGGCCCCGTAATGCAGAAGAAGACCATGGGCTGGGA
GGCCTCCTCCGAGCGGATGTACCCCGAGGACGGCGCCCTGAAGGGCGAGATCAAGCAGAGGCTGAAGCTGAAGGACGGCGGCCACTACGACGCT
GAGGTCAAGACCACCTACAAGGCCAAGAAGCCCGTGCAGCTGCCCGGCGCCTACAACGTCAACATCAAGTTGGACATCACCTCCCACAACGAGG
ACTACACCATCGTGGAACAGTACGAACGCGCCGAGGGCCGCCACTCCACCGGCGGCATGGACGAGCTGTACGGATCCATGGAAGATGCCAAAAA
CATTAAGAAGGGCCCAGCGCCGTTCTACCCACTCGAAGACGGGACCGCCGGCGAGCAGCTGCACAAAGCCATGAAGCGCTACGCCCTGGTGCCC
GGCGCCATCGCCTTTACCGACGCACATATTCAGGTGGACGTTACCTACGCCGAGTACTTCGAGATGAGCGTTCGGCTGGCAGAAGCTATGAGGC
GCTATGGGCTGAATACAAACCATCGGATCGTGGTGTGCAGCGAGAATAGCTCGCAGTTCTTCATGCCCGTGTTGGGTGCCCTGTTCATCGGTGT
GGCTGTGGCCCCAGCTAACGACATCTACAACGAGCGCGAGCTGCTGAACAGCATGGGCATCAGCCAGCCCACCGTCGTATTCGTGAGCAAGAAA
GGGCTGCGAAAGGTCCTCAACGTGCAAAAGAAGCTACCGATCATACGAAAGATCATCATCATGGATAGCAAGACCGACTACCAGGGCTTCCAAA
GCATGTACACCTTCGTGACTTCCCATTTGCCACCCAGCTTCAACGAGTACGACTTCGTGCCCGAGAGCTTCGACCGGGACAAAACCATCGCCCT

GATCATGAACAGTAGTGGTAGTACAGGATTACCCAAGGGCGTAGCCCTACCGCACCGCACCGCTTGTGTCCGATTCAGTCATGCCCGCGACCCC
ATCTTCGGCTACCAGAACATCCCCGACACCGCTATCCTCAGCGTGGTGCCATTTCACCACGGCTTCGGCATGTTCACCACGCTGGGCTACTTGA
TCTGCGGCTTTCGGGTCGTGCTCATGTACCGCTTCGAGGAGGAGCTATTCTTGCGCAGCTTGCAAGACTATAAGATTCAATCTGCCCTGCTGGT
GCCCACACTATTTAGCTGCCTCGCTAAGAGCACTCTCATCGACAAGTACGACCTAAGCAGCTTGCGCGAGATCGCCAGCGGCGGGGCGCCGCTC
AGCAAGGAGGTAGGTGAGGCCGTGGCCAAACGCTTCCGCCTACCAGGCATCCGCCAGGGCTATGGCCTGACAGAAACAACCAACGCCGTCATGA
TCACCCCCGAGGGGGACCGTAAGCCTGGCTCAGTAGGCAAGGTGGTGCCCTTCTTCGAGGCTAAGGTGGTAGACTTGGTCACCGGTAAGACACT
GGGTGTGAACCAGCGCGGTGAGCTGTGCGTCCGTGGCCCCATGATCATGAGCGGCTACGTTAACAACCCCGAGGCTACGAACGCTCTCATCGAC
AAGGACGGCTGGCTGCACAGCGGCGACATCGCCTACTGGGACGAGGACGAGCACTTCTTCATCGTGGACCGGCTGAAGAGCCTGATCAAATACA
AGGGCTACCAGGTAGCCCCAGCCGAACTGGAGGGCATCCTGCTGCAACACCCCTACATCTTCGACGCCGGAGTCGCCGGCCTGCCCGACGACGA
TGCCGGCGAGCTGCCCGCCGCAGTCGTCGTGTTGGAACACGGTAAAACCATGACCGAGAAAGAGATCGTGGACTATGTGGCCAGCCAGGTTACA
ACCGCCAAGAAGCTGCGCGGTGGTGTTGTGTTTGTGGATGAAGTCCCTAGAGGATCGACCGGCAAGTTAGACGCCCGCAAGATCCGCGAGATTC
TCACTAAGGCCAAGAAGGACGGCAAGATCGCCGTGTAAGAAGTCGACGGATCCTCGATAATCAACCTCTGGATTACAAAATTTGTGAAAGATTG
ACTGGTATTCTTAACTATGTTGCTCCTTTTACGCTATGTGGATACGCTGCTTTAATGCCTTTGTATCATGCTATTGCTTCCCGTATGGCTTTCA
TTTTCTCCTCCTTGTATAAATCCTGGTTGCTGTCTCTTTATGAGGAGTTGTGGCCCGTTGTCAGGCAACGTGGCGTGGTGTGCACTGTGTTTGC
TGACGCAACCCCCACTGGTTGGGGCATTGCCACCACCTGTCAGCTCCTTTCCGGGACTTTCGCTTTCCCCCTCCCTATTGCCACGGCGGAACTC
ATCGCCGCCTGCCTTGCCCGCTGCTGGACAGGGGCTCGGCTGTTGGGCACTGACAATTCCGTGGTGTTGTCGGGGAAGCTGACGTCCTTTCCAT
GGCTGCTCGCCTGTGTTGCCACCTGGATTCTGCGCGGGACGTCCTTCTGCTACGTCCCTTCGGCCCTCAATCCAGCGGACCTTCCTTCCCGCGG
CCTGCTGCCGGCTCTGCGGCCTCTTCCGCGTCTTCGCCTTCGCCCTCAGACGAGTCGGATCTCCCTTTGGGCCGCCTCCCCGCATCTCGACAAA
GGATCCAATGGAGGCCGCCACCGCGGTGGAGCTCGAATTAATTCATCGATGATGATCCAGACATGATAAGATACATTGATGAGTTTGGACAAAC
CACAACTAGAATGCAGTGAAAAAAATGCTTTATTTGTGAAATTTGTGATGCTATTGCTTTATTTGTAACCATTATAAGCTGCAATAAACAAGTT
AACAACAACAATTGCATTCATTTTATGTTTCAGGTTCAGGGGGAGGTGTGGGAGGTTTTTTAAAGCAAGTAAAACCTCTACAAATGTGGTATGG
CTGATTATGATCCAAGCTTGGCGTAATCATGGTCATAGCTGTTTCCTGTGTGAAATTGTTATCCGCTCACAATTCCACACAACATACGAGCCGG
AAGCATAAAGTGTAAAGCCTGGGGTGCCTAATGAGTGAGCTAACTCACATTAATTGCGTTGCGCTCACTGCCCGCTTTCCAGTCGGGAAACCTG
TCGTGCCAGCGGATCTTAAAAGTTTTGTTACTTTATAGAAGAAATTTTGAGTTTTTGTTTTTTTTTAATAAATAAATAAACATAAATAAATTGT
TTGTTGAATTTATTATTAGTATGTAAGTGTAAATATAATAAAACTTAATATCTATTCAAATTAATAAATAAACCTCGATATACAGACCGATAAA
ACACATGCGTCAATTTTACGCATGATTATCTTTAACGTACGTCACAATATGATTATCTTTCTAGGGTTAATCTAGTATACGCGTTTAATTAATC
CAGCTTTTGTTCCCTTTAGTGAGGGTTAATTGCGCGCTTGGCGTAATCATGGTCATAGCTGTTTCCTGTGTGAAATTGTTATCCGCTCACAATT
CCACACAACATACGAGCCGGAAGCATAAAGTGTAAAGCCTGGGGTGCCTAATGAGTGAGCTAACTCACATTAATTGCGTTGCGCTCACTGCCCG
CTTTCCAGTCGGGAAACCTGTCGTGCCAGCTGCATTAATGAATCGGCCAACGCGCGGGGAGAGGCGGTTTGCGTATTGGGCGCTCTTCCGCTTC
CTCGCTCACTGACTCGCTGCGCTCGGTCGTTCGGCTGCGGCGAGCGGTATCAGCTCACTCAAAGGCGGTAATACGGTTATCCACAGAATCAGGG
GATAACGCAGGAAAGAACATGTGAGCAAAAGGCCAGCAAAAGGCCAGGAACCGTAAAAAGGCCGCGTTGCTGGCGTTTTTCCATAGGCTCCGCC
CCCCTGACGAGCATCACAAAAATCGACGCTCAAGTCAGAGGTGGCGAAACCCGACAGGACTATAAAGATACCAGGCGTTTCCCCCTGGAAGCTC
CCTCGTGCGCTCTCCTGTTCCGACCCTGCCGCTTACCGGATACCTGTCCGCCTTTCTCCCTTCGGGAAGCGTGGCGCTTTCTCATAGCTCACGC
TGTAGGTATCTCAGTTCGGTGTAGGTCGTTCGCTCCAAGCTGGGCTGTGTGCACGAACCCCCCGTTCAGCCCGACCGCTGCGCCTTATCCGGTA
ACTATCGTCTTGAGTCCAACCCGGTAAGACACGACTTATCGCCACTGGCAGCAGCCACTGGTAACAGGATTAGCAGAGCGAGGTATGTAGGCGG
TGCTACAGAGTTCTTGAAGTGGTGGCCTAACTACGGCTACACTAGAAGGACAGTATTTGGTATCTGCGCTCTGCTGAAGCCAGTTACCTTCGGA
AAAAGAGTTGGTAGCTCTTGATCCGGCAAACAAACCACCGCTGGTAGCGGTGGTTTTTTTGTTTGCAAGCAGCAGATTACGCGCAGAAAAAAAG
GATCTCAAGAAGATCCTTTGATCTTTTCTACGGGGTCTGACGCTCAGTGGAACGAAAACTCACGTTAAGGGATTTTGGTCATGAGATTATCAAA
AAGGATCTTCACCTAGATCCTTTTAAATTAAAAATGAAGTTTTAAATCAATCTAAAGTATATATGAGTAAACTTGGTCTGACAGTTACCAATGC
TTAATCAGTGAGGCACCTATCTCAGCGATCTGTCTATTTCGTTCATCCATAGTTGCCTGACTCCCCGTCGTGTAGATAACTACGATACGGGAGG
GCTTACCATCTGGCCCCAGTGCTGCAATGATACCGCGAGACCCACGCTCACCGGCTCCAGATTTATCAGCAATAAACCAGCCAGCCGGAAGGGC
CGAGCGCAGAAGTGGTCCTGCAACTTTATCCGCCTCCATCCAGTCTATTAATTGTTGCCGGGAAGCTAGAGTAAGTAGTTCGCCAGTTAATAGT
TTGCGCAACGTTGTTGCCATTGCTACAGGCATCGTGGTGTCACGCTCGTCGTTTGGTATGGCTTCATTCAGCTCCGGTTCCCAACGATCAAGGC
GAGTTACATGATCCCCCATGTTGTGCAAAAAAGCGGTTAGCTCCTTCGGTCCTCCGATCGTTGTCAGAAGTAAGTTGGCCGCAGTGTTATCACT
CATGGTTATGGCAGCACTGCATAATTCTCTTACTGTCATGCCATCCGTAAGATGCTTTTCTGTGACTGGTGAGTACTCAACCAAGTCATTCTGA
GAATAGTGTATGCGGCGACCGAGTTGCTCTTGCCCGGCGTCAATACGGGATAATACCGCGCCACATAGCAGAACTTTAAAAGTGCTCATCATTG
GAAAACGTTCTTCGGGGCGAAAACTCTCAAGGATCTTACCGCTGTTGAGATCCAGTTCGATGTAACCCACTCGTGCACCCAACTGATCTTCAGC
ATCTTTTACTTTCACCAGCGTTTCTGGGTGAGCAAAAACAGGAAGGCAAAATGCCGCAAAAAAGGGAATAAGGGCGACACGGAAATGTTGAATA
CTCATACTCTTCCTTTTTCAATATTATTGAAGCATTTATCAGGGTTATTGTCTCATGAGCGGATACATATTTGAATGTATTTAGAAAAATAAAC
AAATAGGGGTTCCGCGCACATTTCCCCGAAAAGTGCCACCTAAATTGTAAGCGTTAATATTTTGTTAAAATTCGCGTTAAATTTTTGTTAAATC
AGCTCATTTTTTAACCAATAGG

Tanaka et al. Fig.S1
