# Supplementary figures and images for: A Dual Promoter System to Monitor IFN-γ Signaling in vivo at Single-cell Resolution
Source: Cell Struct Funct. 2021 Nov 6;46(2):103–11. doi: 10.1247/csf.21052 (PMC10511040; doi:10.1247/csf.21052)

Figure S2

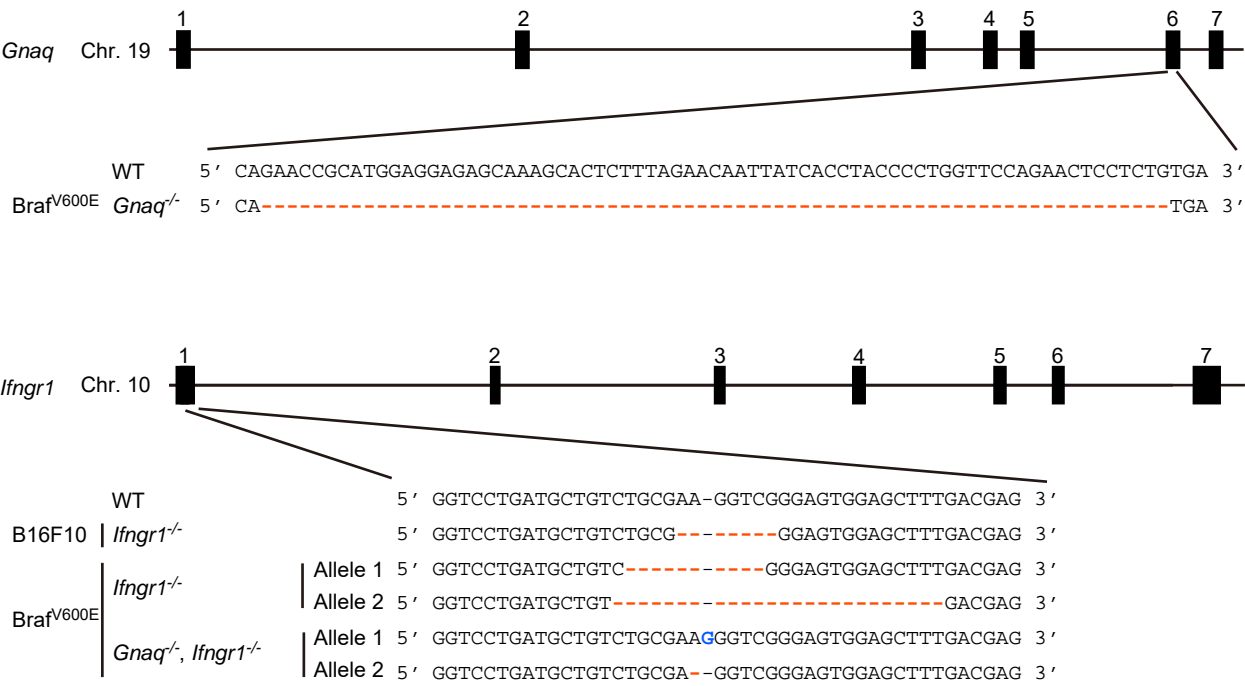

Supplement: Supplementary file 2 — Fig. S2 [file csf_46_21052_2.pdf]
